# Genetic analysis of arsenic accumulation in maize using QTL mapping

**DOI:** 10.1038/srep21292

**Published:** 2016-02-16

**Authors:** Zhongjun Fu, Weihua Li, Xiaolong Xing, Mengmeng Xu, Xiaoyang Liu, Haochuan Li, Yadong Xue, Zonghua Liu, Jihua Tang

**Affiliations:** 1 Key Laboratory of Wheat and Maize Crops Science, Collaborative Innovation Center of Henan Grain Crops, College of Agronomy, Henan Agricultural University, Zhengzhou 450002, China; 2 Maize Research Institute, Chongqing Academy of Agricultural Sciences, Chongqing 401329, China; 3 Hubei Collaborative Innovation Center for Grain Industry, Yangtze University, Jingzhou 434023, China

## Abstract

Arsenic (As) is a toxic heavy metal that can accumulate in crops and poses a threat to human health. The genetic mechanism of As accumulation is unclear. Herein, we used quantitative trait locus (QTL) mapping to unravel the genetic basis of As accumulation in a maize recombinant inbred line population derived from the Chinese crossbred variety Yuyu22. The kernels had the lowest As content among the different maize tissues, followed by the axes, stems, bracts and leaves. Fourteen QTLs were identified at each location. Some of these QTLs were identified in different environments and were also detected by joint analysis. Compared with the B73 RefGen v2 reference genome, the distributions and effects of some QTLs were closely linked to those of QTLs detected in a previous study; the QTLs were likely in strong linkage disequilibrium. Our findings could be used to help maintain maize production to satisfy the demand for edible corn and to decrease the As content in As-contaminated soil through the selection and breeding of As pollution-safe cultivars.

Arsenic (As), which is a highly toxic metalloid and is found ubiquitously in the environment, poses a serious risk to plants, animals and humans^1^. Arsenic content in soils has increased substantially in recent years because of irrigation with As-rich surface water or from anthropogenic activities, such as ore mining, smelting, burning of coal, use of As pesticides and the application of wastes^2^,^3^,^4^. Excess As in soil can inhibit seed germination and plant growth^5^,^6^,^7^, disturb plant metabolism^8^ and cause plant death^9^,^10^. Arsenic can be taken up by plants and vegetables from the soil and irrigated water, and subsequently enter the food chain^11^. Thus, humans may consume As. Arsenic exposure can cause human diseases such as skin lesions, neurological defects, atherosclerosis and even cancer^12^. In recent years, the most serious As pollution problems have occurred in Bangladesh and West Bengal, India^13^,^14^. Therefore, there is an urgent need to find suitable methods to reduce the transmission of As to humans. One option is to remove As from the soil. However, traditional methods and phytoremediation are limited by their own shortcomings^15^,^16^,^17^. Yu *et al.* have described the concept of the pollution-safe cultivar^18^. This concept refers to the use of cultivars that accumulate a very low level of a specific pollutant, which ensures the crop remains safe for human consumption, even when grown in contaminated soil. The application of pollution-safe cultivar selection and breeding is considered a practical and cost-effective approach to minimize the entry of heavy metals into the human food chain, and has received widespread attention^19^,^20^.

Human exposure to As occurs commonly by transfer from the crop–soil–water system^1^. Recently, the accumulation and distribution of As have been studied in different crops. Abedin *et al.* found that rice roots accumulated much more As than the straw and grain^21^. The trend of As concentration in different rice tissues was as follows: grain <husk <straw <root^22^. In maize, Baig *et al.* reported that the total As content in different tissues was in the order: grain <shoot <root^23^. Other studies have reported that the trend of As concentration in four different maize tissues was: kernels <bracts <stems <leaves^24^,^25^.

Marker-assisted selection is very useful to accelerate genetic improvement in crops. Many quantitative trait loci (QTLs) for important traits have been mapped in whole genomes^26^, forming the basis for rapid genetic improvement through marker-assisted selection. Four QTLs for As accumulation have been detected in rice^27^, and Tapash *et al.* identified an As tolerance gene on chromosome 6 of rice using a recombinant inbred line (RIL) population from a Bala × Azucena population^28^. In maize, many studies have focused on the physiological and biochemical responses to As accumulation. The majority of these studies demonstrated a trend of decreasing As content from the roots to the aerial parts, including the leaves, stems and seeds^1^. Maize takes up the arsenic naturally present in the soil or arsenic that is added through groundwater irrigation or by soil additives contaminated with arsenic. Several studies have described a significant relationship between the As concentration in the irrigation water or soil and the total As content accumulated by maize plants^29^. Gulz *et al.* observed that the correlation between the total accumulated As in maize plants and the water-soluble As fraction in the soil was higher than the total As content in the soil^30^. Several factors, including pH, redox potential, organic matter content, interaction/competition with other elements and chemical forms of the pollutant, can affect As solubility in soils^31^.

Maize is the most cultivated cereal in the world and is used as an important animal feed or a staple food crop for humans in many developing countries in Africa, Asia and Latin America^1^. Hence, maize grown on As-contaminated land could accumulate As and pose a risk to human health. Thus, methods to reduce As accumulation in maize are urgently needed. However, there have been few reports of mapped QTLs associated with As accumulation and distribution in maize. In the present study, a RIL population derived from parents with contrasting As tolerances was studied at two locations where the soil As levels substantially differed. The accumulation and distribution of As in different maize tissues were examined. Additionally, we generated data that may aid QTL mapping for important traits in breeding populations for the genetic improvement and production of maize pollution-safe cultivars.

## Results

### Performance of the measured traits at two locations

**Figure 1:**
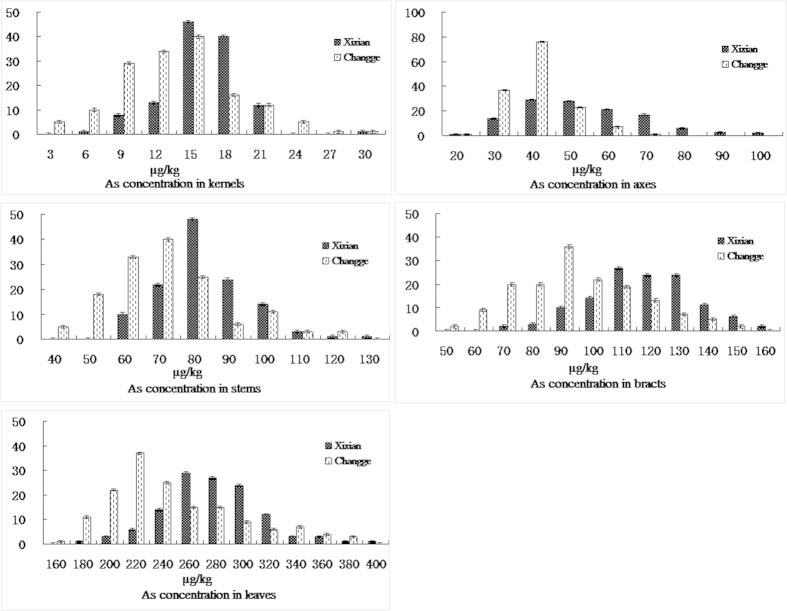
Histogram of As concentration in five maize tissues of the recombinant inbred line (RIL) population.

The soil As concentration at Xixian was 20.70 ± 0.37 mg/kg (pH = 6.5) because of irrigation with As-rich surface water. While the soil As concentration at Changge was 12.24 ± 0.21 mg/kg (pH = 6.5), which was used as a control. In terms of As concentration of the two parents and the hybrid, the five measured tissues showed higher levels at Xixian compared with Changge (Table 1, Fig. 1). The As content in the five maize tissues examined varied widely in the RIL population, which showed two-way transgressive segregation. In addition, the average As concentration in the RIL population was higher at Xixian than at Changge. The results indicated that soil As concentration is an important factor affecting the As content in maize tissues.

### Variance analysis of As accumulation in different maize tissues

The trend of As concentration in different maize tissues at the two locations was as follows: kernels <axes <stems <bracts <leaves. Analysis of variance indicated the As concentration in the five measured tissues in the RIL population was significantly affected by environment, block, genotype and genotype-by-environment factors (Table 2). The As concentration in the kernels largely differed by genotype and genotype-by-environment factors, explaining 32.03% and 39.86% of the total variation, respectively. However, the environment and block factors only contributed 8.43% and 1.16% of the total variation, respectively. The same trend was observed for the As concentration in the axes, stems, bracts and leaves. These results indicated that the differences in As concentrations in different maize tissues mostly depended on the genotype and the interaction between the genotype and the environment. The Pearson correlation coefficient was used to calculate the correlation of As content among the different tissues. The results showed that there were no significant relationships among the As concentration in the five measured tissues at the two locations (Table 3).

### QTL analysis of As content in different maize tissues

#### QTLs identified in the two locations

**Figure 2:**
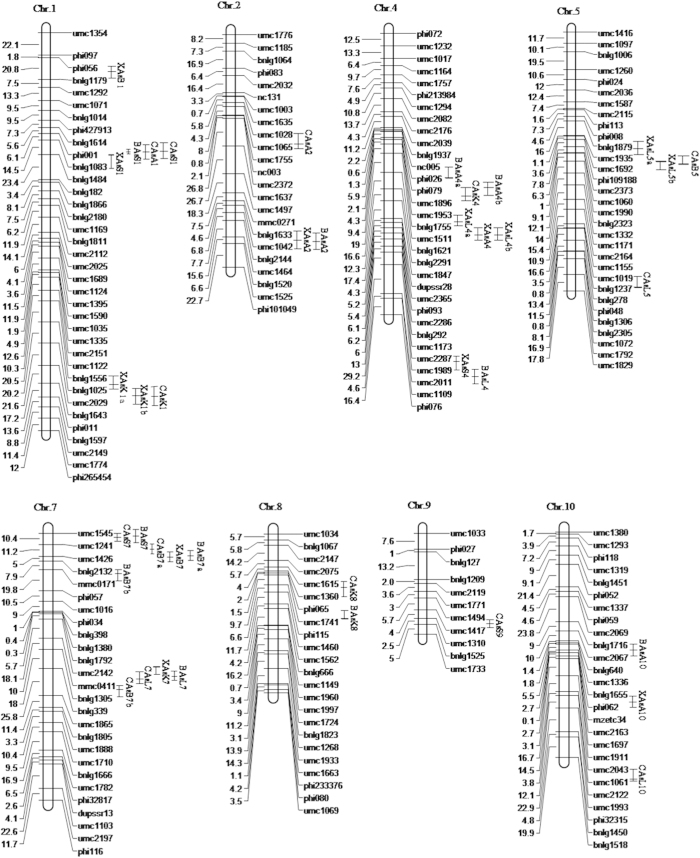
Chromosomal locations of quantitative trait loci (QTLs) for As concentration in five maize tissues.

Twenty-eight QTLs related to the As concentration in the different maize tissues were detected in plants at the two locations (Table 4, Fig. 2). The QTLs were found on all chromosomes, except chromosomes 3 and 6. Six QTLs for the kernel As concentration (KAC) were identified at both locations. Two QTLs, *XAsK1b* and *CAsK1*, were detected in the common regions in the interval 220.20M–233.37M on chromosome 1. *XAsK1a* was identified at Xixian, which explained a relatively large proportion (26.50%) of the variance. For the six QTLs for KAC, the increasing effects of alleles came from both parents. Five QTLs were identified for the axes As concentration (AAC) at the two locations. However, there were no stable QTLs for AAC at the two locations. Of the five QTLs for AAC, three had positive additive effects, indicating that Zong3 contributed more to the AAC. Two QTLs for the stem As concentration (SAC), *XAsS1* and *CAsS1*, were adjacent to each other on chromosome 1. All five identified QTLs for SAC had positive additive effects, which indicated that Zong3 contributed more to the SAC. For the bract As concentration (BAC), five QTLs were detected at the two locations. For *XAsB7* and *CAsB7a*, one common region was found in the interval 2.27M–2.91M on chromosome 7. Four of the five QTLs for BAC had negative additive effects, which indicated that 87-1 contributed more to the BAC. Seven QTLs for the leaf As concentration (LAC) were identified at the two locations. Stable QTLs were not observed in the different environments. For these QTLs, the increasing effects of the alleles came from both parents.

#### QTLs identified by best linear unbiased predictions

Eleven QTLs were detected in a joint analysis of the two locations (see the best linear unbiased predictions (BLUP) section of Table 4). Six QTLs including one for the axes, three for the stems, one for the bracts and one for the leaves were also detected in a single environment analysis in common marker intervals. Additionally, some QTLs detected in the joint analysis were not detected in the single environment analysis (e.g., *BAsK8*, *BAsA4a*, *BAsA10*, *BAsB7b* and *BAsL4*). These may have been only minor QTLs and were not stable across different environments.

## Discussion

In the present study, we found that As levels in maize tissues followed the trend: leaves >bracts >stems >axis >kernels. Regarding environmental As content, Gulz *et al.* observed that the As content in maize roots was positively correlated with the total As content in the soil^30^. In a study conducted in Thailand, Prabpai *et al.* reported that there was a direct linear relationship between As accumulation in maize tissues and the total soil As content^29^. Additionally, in the present study, we observed that the maize tissue As concentrations were significantly affected by the genotype, environment and genotype-by-environment interactions. However, there were small environment-related differences in the As concentrations in the various maize tissues, and the genotype and genotype-by-environment interactions contributed more to the total variation. These results suggest that As concentration in different maize tissues could be reduced through genetic improvement.

To the best of our knowledge, only one article has reported QTLs related to As accumulation in different maize tissues. Ding *et al.* detected 11 QTLs for As accumulation in four different maize tissues^24^. In our study, 14 QTLs were identified at each location and 11 QTLs were identified in a joint analysis. Some of these QTLs were identified in different environments and were also detected by joint analysis. Compared with the B73 RefGen v2 reference genome, the distributions and effects of certain QTLs were closely linked to those of QTLs detected in previous studies. Most QTLs clustered at 27.92 M–46.38 M (chromosome 1), 209.87 M–233.37 M (chromosome 1), 75.51 M–118.32 M (chromosome 4), 1.56 M–2.91 M (chromosome 7) and 108.54 M–131.01 M (chromosome 7). *XAsK1b* and *CAsK1* were detected in a common chromosomal region (209.87 M–233.37 M in chromosome 1) and were clustered with *XAsK1a*. Within the same genomic region, Qin *et al.* detected a QTL for Zn content in maize kernels using an F_2:3_ population^32^. Additionally, Liu *et al.* found two QTLs related to drought tolerance using a RIL population derived from the parents, Zong3 and 87-1^33^. *XAsL4a* was found in common regions with a QTL for Hg accumulation, which had been identified using the same population in a previous study^34^. There may be strong linkage disequilibrium between QTLs and a genome-wide association study (GWAS) will be conducted to test the linkage disequilibrium in the future. Our results and those reported in previous studies identified certain chromosomal regions that should be analyzed further. These regions may represent targets for marker-assisted selection of maize cultivars with low As concentrations.

In maize, kernels are the main edible parts for humans and animals. In this study, the kernels contained the lowest As concentration, while the main biomass products including the leaves, bracts, stems and axes had relatively high As concentrations. Additionally, the As concentration in the kernels was considerably lower than the limit of 200 μg/kg specified in the National Standard of China (GB2762-2005). Maize is capable of adapting to its environment and is widely planted globally. Therefore, it is important to ensure maize production continue to satisfy the global demand for edible corn and to decrease the As content in As-contaminated soil by selecting and breeding As pollution-safe cultivars.

## Materials and Methods

### Experimental locations

The field experiments were conducted in 2012 at Xixian (E114° 72′, N32° 35′) and Changge (E113° 34′, N34° 09′) counties, which are located in northern China, with average temperatures of 15.2 °C and 14.3 °C, respectively, and rainfalls of 873.8 mm and 462.8 mm, respectively.

### Plant materials

A mapping population of 194 F8 generation RILs was used. The RILs were derived from a cross between inbred line Zong3 (from a synthetic population of Chinese domestic germplasm) and 87-1 (from an exotic germplasm) using a single seed descent method. The RIL population, the two parents and the hybrid (Yuyu22) were grown in 2012 at Xixian and Changge using a randomized complete block design, with three replications at each location. Each plot included 15 plants with one 6 m × 0.67 m row, allowing a density of 45,000 plants per hectare.

### Determination of the As concentration in maize

All plant materials were harvested when they reached physiological maturity and five consecutive plants from each plot were selected for further analysis. Mature plants were dissected into five parts: kernels, axes, stems, bracts and leaves. The collected plant materials were dried and ground into fine powder using a mortar and pestle. Powdered samples (0.5 g) were added to polypropylene tubes and digested with 5 mL HNO_3_/HClO_4_ (80/20 v/v) using a heating block (AIM500 Digestion System, A.I. Scientific, Australia). The concentrations of As in the different plant materials were then determined using atomic fluorescence spectrometry (AFS-3000, Beijing Haiguang Analytical Instrument Co., Beijing, China).

### Statistical analysis

The expected genotypic variance (G), block variance (B), environmental variance (E) and G × E interaction were estimated by two-way ANOVA using the IBM SPSS Statistics package. The broad-sense heritabilities were calculated for genotype variance of each effect by the total sum of genotype variance and environmental variance. In the present study, 194 F8 generation RILs, which were derived from a cross between inbred line Zong3 and 87-1, were used as the materials. The two parents have contrasting levels of As accumulation. The soils of the test locations also have contrasting As contents. The materials and test locations were selected such that the G, E, and G × E terms were fitted as a fixed effect.

### Linkage map construction and QTL analysis

The genetic linkage map, consisting of 263 simple sequence repeat markers, was constructed using Mapmaker 3.0 and covered 2,361 cM, with an average interval of 9 cM between markers^35^. The QTLs were identified with Model 6 of the Zmapqtl module of QTL Cartographer 2.5 using the composite interval mapping method^36^. The logarithm of odds thresholds for all measured traits were calculated by 1,000 random permutations at a significance level of α = 0.05, scanning intervals of 2 cM between markers and putative QTLs, with a 10 cM window^37^. The number of marker cofactors for background controls was determined by stepwise regression with five controlling markers. The phenotypic data for each measured material were based on the average values of three replicates. BLUP of arsenic concentration for each plant material at the two locations was calculated by a random effects model using the MIXED procedure in SAS. The QTLs were annotated as follows: for example, for *XAsK1a*, the “X” indicates the location at which the QTL was detected (Xixian, Changge and BLUP were abbreviated as X, C and B, respectively), “AsK” represents the arsenic concentration of the kernels (axes, stems, bracts and leaves were abbreviated as A, S, B and L, respectively), the number “1” is the serial number of the chromosome and “a” represents the serial number of the identified QTL.

## Additional Information

**How to cite this article**: Fu, Z. *et al.* Genetic analysis of arsenic accumulation in maize using QTL mapping. *Sci. Rep.* **6**, 21292; doi: 10.1038/srep21292 (2016).

## Figures and Tables

**Table 1:** Performance of five maize tissues in the RIL population.

Location	Population	Trait	KAC (μg/kg)	AAC (μg/kg)	SAC (μg/kg)	BAC (μg/kg)	LAC (μg/kg)
Xixian	Zong3	Mean	11.54 ± 0.87	28.20 ± 1.18	96.63 ± 7.12	141.41 ± 10.50	239.59 ± 16.55
	87-1	Mean	18.22 ± 1.45	53.17 ± 2.18	69.18 ± 1.12	99.62 ± 4.88	296.14 ± 17.58
	F1	Mean	14.27 ± 0.55	78.95 ± 5.46	79.00 ± 5.50	113.81 ± 5.05	328.17 ± 20.53
	RIL	Mean	14.66 ± 3.48	47.59 ± 16.12	77.08 ± 12.05	112.08 ± 18.07	268.02 ± 36.18
		Range	5.66–29.00	19.81–97.70	52.35–123.99	60.20–155.40	170.21–384.49
		Skewness	0.38	0.69	0.65	−0.23	0.33
		Kurtosis	1.94	0.19	1.36	0.06	0.82
Changge	Zong3	Mean	8.63 ± 1.02	26.85 ± 3.96	67.28 ± 2.80	50.00 ± 2.26	260.44 ± 10.39
	87-1	Mean	18.75 ± 2.74	39.87 ± 4.95	41.68 ± 7.20	78.48 ± 1.45	212.53 ± 10.32
	F1	Mean	13.07 ± 3.39	32.48 ± 1.53	66.05 ± 8.90	98.03 ± 4.34	242.03 ± 12.33
	RIL	Mean	12.03 ± 4.88	35.04 ± 7.47	66.44 ± 16.80	89.62 ± 21.17	237.73 ± 50.19
		Range	0.88–29.38	21.57–64.69	35.91–114.68	47.67–142.74	152.04–377.28
		Skewness	0.46	1.14	0.79	0.38	0.81
		Kurtosis	0.53	1.58	0.47	−0.30	0.08

KAC: kernel As concentration, AAC: axis As concentration, SAC: stem As concentration, BAC: bract As concentration, LAC: leaf As concentration, RIL; recombinant inbred line.

**Table 2:** Variance analysis of the five measured tissues in the RIL population.

Tissue	Variance	MS	F	SS/SST^a^	p value^d^	h^2^b (%)^c^
Kernel	B	93.66	13.66^**^	1.16	1.72E–06	71.9
	L	1363.24	210.35^**^	8.43	1.5E–39	
	G	45.01	6.95^**^	32.03	6.3E–52	
	L × G	56.02	8.64^**^	39.86	1.49E–64	
Axis	B	103.19	6.10^**^	0.24	0.002	85.8
	L	17375.41	1050.62^**^	9.74	1.7E–121	
	G	639.64	38.68^**^	41.58	3.9E–181	
	L × G	680.85	41.17^**^	44.25	9.8E–187	
Stem	B	183.47	6.70^**^	0.14	0.001	88.7
	L	13220.71	495.85^**^	5.92	5.12E–74	
	G	1046.41	39.25^**^	51.04	1.3E–171	
	L × G	771.82	28.95^**^	37.65	1.7E–146	
Bract	B	432.05	16.16^**^	0.22	1.63E–07	69.3
	L	106736.17	4267.72^**^	27.42	5.9E–238	
	G	1089.78	43.57^**^	32.76	1.7E–193	
	L × G	1216.58	48.64^**^	36.57	1.3E–203	
Leave	B	198.84	4.00^*^	0.03	0.019	90.0
	L	121193.22	2469.75^**^	8.35	3.1E–189	
	G	5509.44	112.27^**^	44.39	2.3E–283	
	L × G	5665.05	115.45^**^	45.65	4.4E–286	

**significant at α = 0.01, *significant at α = 0.05.

E: environment, B: block, G: genotype, G × E: genotype-by-environment interaction.

^a^Sum of squares (SS) of each effect by total SS (SST).

^d^p value, statistical significance of five five measured tissues in the two locations.

^c^h^2^b, broad-sense heritability.

RIL; recombinant inbred line.

**Table 3:** Correlation coefficients among five maize tissues in the RIL population.

Location	Trait	Kernel	Axis	Stem	Bract	Leave
Xixian	Kernels	1.00				
	Axis	−0.12	1.00			
	Stem	−0.15	−0.06	1.00		
	Bract	0.10	−0.03	0.10	1.00	
	Leaves	0.04	0.07	0.14	0.10	1.00
Changge	Kernels	1.00				
	Axis	−0.15	1.00			
	Stem	−0.14	−0.02	1.00		
	Bract	0.08	−0.03	0.07	1.00	
	Leaves	0.04	0.05	0.13	0.07	1.00
BLUP	Kernels	1.00				
	Axis	0.04	1.00			
	Stem	0.07	0.11	1.00		
	Bract	−0.01	−0.05	−0.06	1.00	
	Leaves	0.06	−0.13	0.10	−0.08	1.00

RIL; recombinant inbred line.

**Table 4:** QTLs detected for As concentration in five maize tissues.

Location	Trait	QTL^a^	Location	Confidence interval	Flanking-markers	Support interval	LOD^b^	A^c^	R^2^^d^
Xixian	Kernel	XAsK1a	275.41	270.41–278.41	bnlg1556–bnlg1025	209.87M–220.20M	3.24	−1.87	26.50
		XAsK1b	282.91	281.91–285.91	bnlg1025–umc2029	220.20M–233.37M	2.85	−1.77	23.93
		XAsK7	81.21	81.21–84.21	umc2142–mmc0411	108.54M–126.46M	2.77	0.98	7.86
	Axis	XAsA2	159.51	156.01–162.51	bnlg1633–umc1042	199.17M–202.21M	4.33	−7.09	12.06
		XAsA4	114.81	109.51–117.81	bnlg1755–umc1511	81.19M–118.32M	3.13	−7.05	12.01
		XAsA10	108.41	106.61–110.41	bnlg1655–phi062	85.27M–102.32M	2.82	6.10	8.83
	Stem	XAsS1	103.51	103.41–112.51	phi001–bnlg1083	27.92M–46.38M	3.93	5.59	11.21
		XAsS4	218.71	214.71–225.71	umc2287–umc1989	230.38M–231.90M	4.76	5.13	14.06
	Bract	XAsB1	36.91	32.91–42.91	phi056–bnlg1179	2.19M–4.99M	3.29	7.50	32.44
		XAsB7	20.41	18.41–22.61	umc1241–umc1426	2.27M–2.91M	2.66	−4.65	9.95
	Leave	XAsL4a	108.51	106.41–109.51	umc1953–bnlg1755	75.51M–118.32M	2.95	−11.54	9.95
		XAsL4b	115.81	111.81–118.81	bnlg1755–umc1511	81.19M–118.32M	2.82	−12.20	11.19
		XAsL5a	103.21	97.21–108.21	bnlg1879–umc1935	14.27M–49.68M	2.93	14.13	14.69
		XAsL5b	114.31	114.21–116.31	umc1935–umc1692	49.68M–53.91M	3.00	10.97	8.82
Changge	Kernel	CAsK1	280.91	272.41–288.91	bnlg1025–umc2029	220.20M–233.37M	3.12	−3.46	12.30
		CAsK4	103.51	101.5–104.41	phi079–umc1896	41.83M–65.70M	2.79	−2.70	7.59
		CAsK8	34.41	32.41–37.41	umc1615–umc1360	71.28M–90.71M	2.82	2.68	7.37
	Axis	CAsA1	96.81	92.81–100.41	bnlg1614–phi001	13.51M–27.92M	3.72	2.81	10.36
		CAsA2	65.01	62.21–66.01	umc1028–umc1065	150.12M–151.45M	3.52	2.32	9.03
	Stem	CAsS1	93.81	89.51–96.81	bnlg1614–phi001	13.51M–27.92M	2.94	6.26	8.57
		CAsS7	9.01	6.01–12.41	umc1545–umc1241	1.56M-1.81M	2.68	6.15	8.32
		CAsS9	55.21	54.51–56.21	umc1494–umc1417	134.30M–134.38M	2.57	6.00	6.81
	Bract	CAsB5	114.21	113.21–114.31	umc1935–umc1692	49.68M–53.91M	3.39	−8.40	8.46
		CAsB7a	15.41	13.41–16.41	umc1241–umc1426	2.27M–2.91M	2.65	−7.43	11.41
		CAsB7b	109.31	107.31–113.31	mmc0411–bnlg1305	126.46M–131.01M	3.02	6.12	7.32
	Leave	CAsL5	211.11	208.51–211.1	umc1019–bnlg1237	191.23M–192.73M	2.68	13.09	6.70
		CAsL7	87.21	78.51–92.21	umc2142–mmc0411	108.54M–126.46M	3.29	−22.55	19.90
		CAsL10	141.21	133.51–143.21	umc2043–umc1061	135.38M–139.10M	2.72	17.81	11.53
BLUP	Kernel	BAsK8	37.41	36.41–37.41	phi065–umc1741	61.30M–70.97M	2.63	0.93	6.41
	Axis	BAsA4a	97.21	95.41–97.21	phi026–phi079	36.88M–41.83M	2.96	−2.48	7.51
		BAsA4b	101.51	100.51–103.51	phi079–umc1896	41.83M–65.70M	2.57	−2.71	8.96
		BAsA10	92.21	87.21–97.21	bnlg1716–umc2067	58.13M–62.25M	3.99	3.70	16.05
	Stem	BAsS1	100.41	95.81–103.51	phi001–bnlg1083	27.92M–46.38M	5.32	4.72	13.77
		BAsA2	160.51	158.01–162.51	bnlg1633–umc1042	199.17M–202.21M	3.16	3.54	7.37
		BAsS7	7.01	1.01–14.41	umc1545–umc1241	1.56M–1.81M	3.84	4.31	11.44
	Bract	BAsB7a	19.41	17.41–21.61	umc1241–umc1426	2.27M–2.91M	2.76	−4.71	12.36
		BAsB7b	31.61	29.61–33.61	bnlg2132–mmc0171	3.25M–4.69M	2.70	−4.31	10.57
	Leave	BAsL4	256.71	250.71–261.91	umc1989–umc2011	230.38M–237.55M	2.78	−14.15	13.19
		BAsL7	85.21	80.51–89.21	umc2142–mmc0411	108.54M–126.46M	2.70	−13.10	11.45

^a^Quantitative trait loci (QTLs) detected for As concentration in five maize tissues.

^b^Logarithm of odds for each QTL.

^c^Additive effect; positive values indicate that Zong3 alleles increase the rates.

^d^R^2^: contribution ratio.
